# Case Report: Modified endoscopic hook for extracting magnetic esophageal foreign bodies in a rural area

**DOI:** 10.12688/f1000research.129564.3

**Published:** 2024-07-25

**Authors:** Hamsu Kadriyan, Ika Primayanti, Syamsidar Syamsidar, Lalu Fauzan Fakhrussiam, Moh Fahrur Rozi, Hijrinelly Hijrinelly

**Affiliations:** 1 Otolaryngology Head and Neck Surgery, Faculty of Medicine, University of Mataram, Indonesia, West Nusa Tenggara, 83125, Indonesia; 2 Public Health, Faculty of Medicine, University of Mataram, Mataram, West Nusa Tenggara, 83125, Indonesia; 3 Nursing Surgery, West Nusa Tenggara Hospital, Mataram, West Nusa Tenggara, 83126, Indonesia; 4 Anesthesiology and reanimation, Faculty of Medicine, University of Mataram, Mataram, West Nusa Tenggara, 83125, Indonesia

**Keywords:** Foreign bodies, aerodigestive tract, endoscopic hook, esophagoscopy

## Abstract

Foreign bodies (FB) in the aerodigestive tract are common, especially in children. The type of foreign body in the esophagus can vary and include magnets. A magnet that lodges in the esophagus should be extracted within 24 hours to prevent complications due to associated chemical reactions. In rural areas, there are several limitations to extracting FBs from the esophagus. We report a case of a magnetic FB that lodged in the esophagus of a three-year-old boy. The extraction was successfully done by esophagoscopy with the modification of a hook that was attached to the endoscope. This innovation may help physician all over the world, especially in rural areas. In the future, this innovation could be produced on an industrial scale.

## Introduction

Although a warning regarding choking risk has been included on every toy by the factory, ^1^ cases of foreign body (FB) in the upper aerodigestive tract remain frequently found in the clinical setting. Several types of FBs can be found in the upper aerodigestive tract, including coins, magnets, batteries, pins, and organic substances such as peanuts, meat, among others. ^2^^–^^4^ The effect of FBs in the aerodigestive tract varies, depending on its location. If the FB is lodged in the esophagus, it will cause dysphagia, while in the bronchus it may cause airway obstruction and lead to mortality. ^2^^,^^3^

The part of a toy such as a magnet can usually found in children and may cause perforation or fistula on the esophagus. This complication occurs because of the effect of the chemical reaction between the magnet with the esophagus tissue. Therefore, a magnet FB in the esophagus should be extracted within 24 hours of the finding. ^3^^–^^5^

The extraction of FBs from the esophagus may be challenging, especially in a rural area with limited equipment. An endoscope or esophagoscope, and a forceps or extractor are not complete or do not fit with the type or shape of the FBs. Therefore, in rural areas, the physician should try to do their best to help the patient with those limitations. In this report, the authors would like to share the modification of the hook that is attached to the rigid endoscope. This modification successfully extracted a big and thick heart-shaped magnet in the esophagus.

## Case presentation

A three-year-old boy was referred from the primary hospital with FBs lodged in the esophagus 1 hour prior to the hospitalization. He accidentally ingested the magnet toys while playing with his sister. After the incident, the boy was crying and his sister told their mother that her brother had ingested the toy part. His sister showed the shape of FB that was ingested to their mother. Therefore, the boy was then brought to the hospital to see whether the FB was lodged somewhere or not. There was no sign of coughing or dyspnea in this patient.

**Figure 1.**
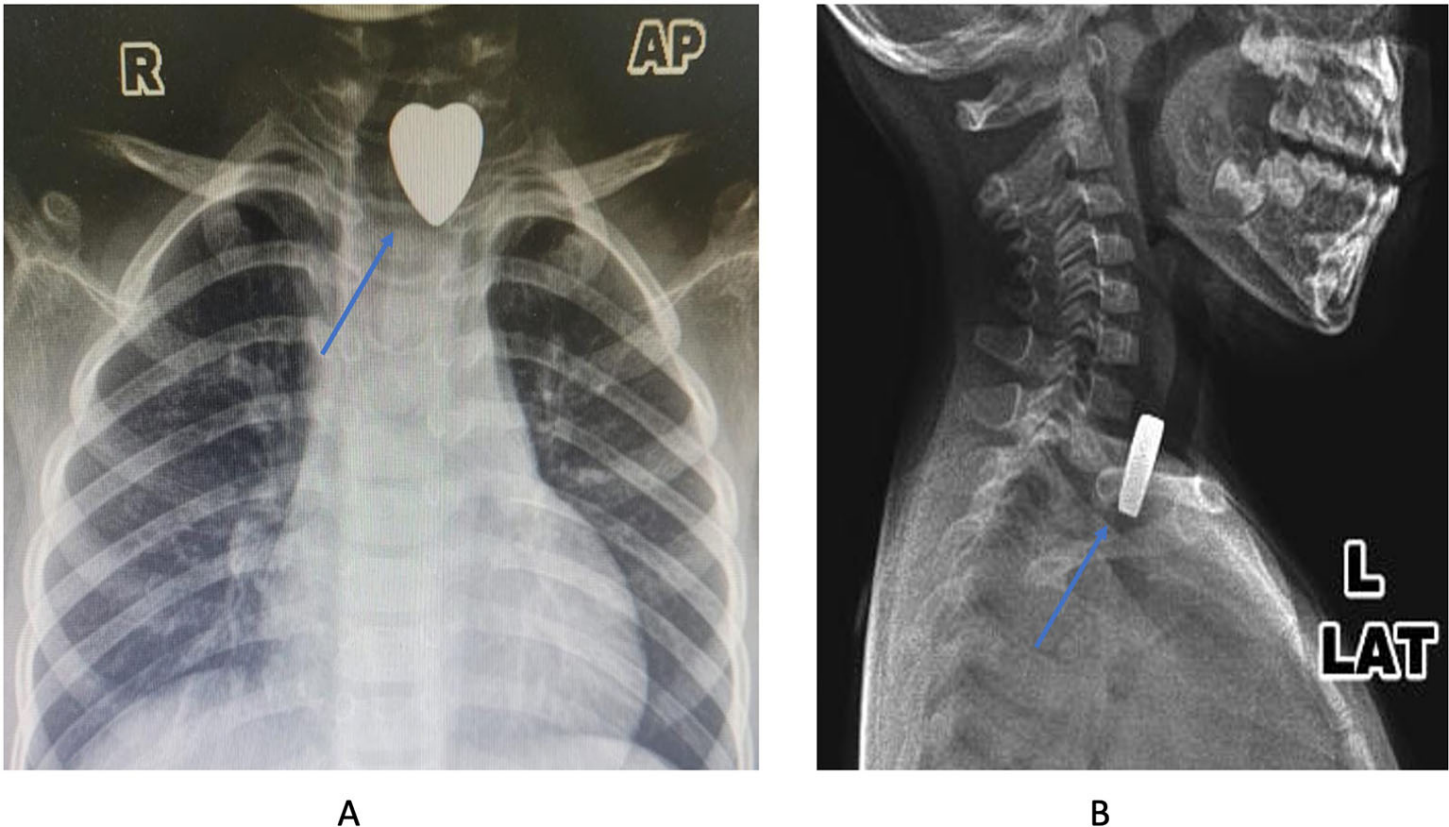
The X-ray images showed the metallic density with the heart shape (blue arrow) (A: anterior-posterior projection; B: lateral projection).

An x-ray examination was done to know the location of the FBs in the upper aerodigestive tract. The result showed the radiopaque metallic object with a heart shape was lodged in the upper esophagus ( Figure 1A and B). After the diagnosis was established, the patient was scheduled for an esophagoscopy to extract the FB under general anesthesia.

**Figure 2.**
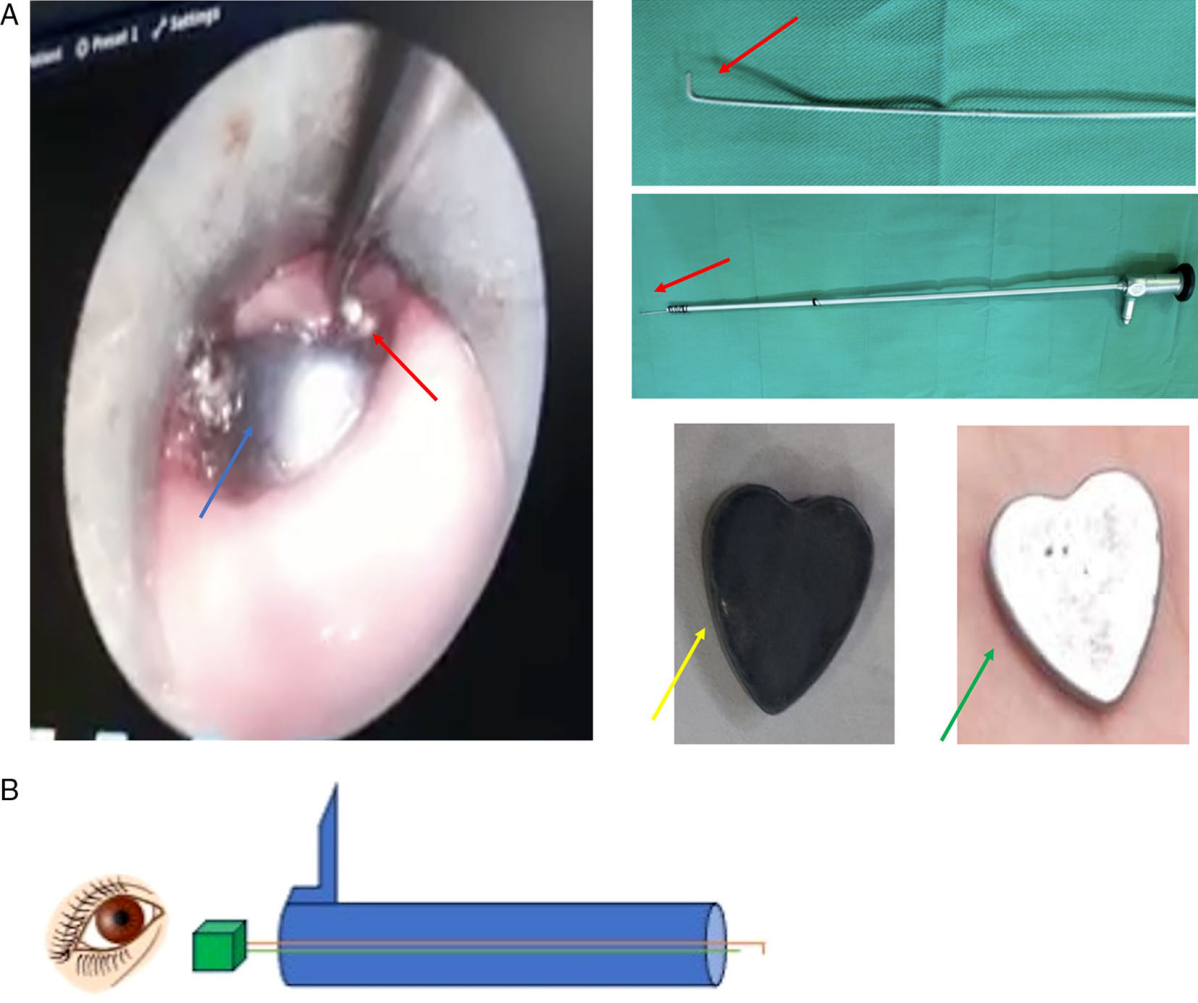
A. The FB extraction under endoscope view (blue arrow: the FB; red arrow: the hook; green arrow: original magnet). B. Schematic design of rigid esophagoscope (blue tubular) with modified hook (brown line) that attached to rigid scope (green line).

During the esophagoscopy, several forceps and baskets were tried to extract the FB, however, the FB was moving down to the middle part. After several attempts, the authors then tried to modify the endoscope by placing the additional hook on the tip of the rigid scope ( Figure 2A, red arrow). Finally, the FB could be extracted without any complications, however, the color of the FB has changed ( Figure 2A, yellow arrow) from its original color ( Figure 2A, green arrow).

Follow-up after surgery was done after two (2) days, and no complications occurred. The patient was then released from the ward. A week after the surgery, the patient came to the outpatient clinic. No difficulties in swallowing were found, and the patient could eat all kinds of food.

## Discussion

The constraints in managing certain cases in rural areas may be unexpected; therefore, some physicians may refer the patient to a bigger/higher hospital if they encounter difficulties. In some archipelago countries, the transportation between the islands may become a problem. Some small islands may only have boating infrastructure; on the other hand, larger islands may have a more complete transportation infrastructure. In some cases, the patient or their family may not agree to be referred to another hospital on other islands. Another patient may delay the treatment time for another reason, for instance, costs considerations and low awareness of disease symptoms. ^6^^,^^7^ As such, the diseases may develop into a more dangerous condition. In the gastrointestinal magnetic FB, it could promote a fistula or perforation. ^5^ Therefore, the physician should apply their best competencies to manage the cases. On the other hand, the patients safety must be a priority. One of the general constraints faced in rural areas is inadequate equipment. The other constraint is manpower. ^7^ The hospital level in some countries is divided into primary to tertiary. Primary hospitals usually have simple equipment and manpower. On the other hand, tertiary hospitals have complete equipment and manpower. ^8^ In Indonesia in 2021 there were only 16 of 34 provinces who have tertiary hospitals, the rest were primary and secondary hospitals. Mostly, the provinces that have tertiary hospitals are located on the big island such as Java island. ^9^

Anatomically, there are several narrow points in the esophagus, consecutively upper, middle, and lower as the common site of FB lodging. The most common lodging site is upper part of esophagus. ^10^ In a rural area such as Africa and Spain, the use of rigid esophagoscope/bronchoscope remain the major procedure for managing FB in aerodigestive tracts. ^11^^,^^12^ This condition is similar to the condition in our region, rigid esophagoscope remains used to extract the FB in the upper digestive tract. Our island is a small island and the distance to the nearest island which has a better health facilities is 4-5 hours by boat.

The modification of this hook was inspired by the cerumen hook that is routinely used by the otolaryngologist to remove the hard wax or FB in the ear canal. ^13^ Therefore, the principle is alike to a cerumen hook. To make it visible, the hook was attached to the rigid scope. Therefore, the FB extraction could be done safely with direct vision. The other concern is the hook should be in a safe mode for the esophagus, with non-sharp tip for example. The schematic design of this equipment ( Figure 2B) could be used to understand it’s properties.

The extraction itself started with the insertion of a rigid esophagoscope into the esophagus until it was close to the FB location. Then, the hook attached to the scope was inserted through the lumen of the esophagoscope. The hook insertion should be done smoothly in a similar direction to the FB position and esophagus wall ( Figure 2A) to prevent a wound that may provoke a perforation. After passing the edge of the FB, the hook direction was then rotated to the body of FB and extracted slowly. If it does not work on the first attempt, the procedure could be repeated until the FB is successfully removed. However, if it is remain fail, the patient should be refer to a better referral health center.

The application of a hook is suitable for a quadrangle- or triangle-shape FB with a certain thickness (thicker than a coin). According to a previous publication, a hook for extracting FB in the esophagus is not available. ^4^ Therefore, this modification is the first innovation of the kind published in a journal. Hopefully, this innovation could help physicians globally, especially in rural areas. This innovation may also be used as an inspiration to modify incomplete equipment in a rural area. However, advanced research should be done to prove it’s safety and efficacy profile. Therefore, in the future, this modification could be produced on an industrial scale.

## Conclusions

The modified endoscopic hook is an innovation that is suitable and safe for extracting quadrangle or triangle FB shapes in the esophagus with a certain thickness.

## Consent of the patient’s parents

The written consent for using the images and publishing this case report has been given by the parent of the patient.
