# Supplementary material for: Case Report: Modified endoscopic hook for extracting magnetic esophageal foreign bodies in a rural area
Source: F1000Res. 2024 Jul 25;12:7. Originally published 2023 Jan 4. [Version 3] doi: 10.12688/f1000research.129564.3 (PMC11342022; doi:10.12688/f1000research.129564.3)
Supplement: Peer review history

# Reviewer report: Reviewer response for version 3

**DOI:** 10.5256/f1000research.169472.r306971

**Published:** 2024-08-16

**Authors:** Ali Bilal Ulas

**Affiliations:** 1 Ataturk University, Erzurum, Turkey

It is erroneous to assume that the fact the procedure is performed under optical observation makes it safe. There is a risk of sudden shifts in the hook and serious injuries during manipulation of the object. As this field is not within the authors' own field of expertise, they are unable to grasp the seriousness of the work. Such risky interventions cannot be considered as innovations in the field of oesophageal surgery.

Are enough details provided of any physical examination and diagnostic tests, treatment given and outcomes?

Partly

Is the case presented with sufficient detail to be useful for other practitioners?

No

Is sufficient discussion included of the importance of the findings and their relevance to future understanding of disease processes, diagnosis or treatment?

No

Is the background of the case’s history and progression described in sufficient detail?

Partly

Reviewer Expertise:

Thoracic Surgery

I confirm that I have read this submission and believe that I have an appropriate level of expertise to state that I do not consider it to be of an acceptable scientific standard, for reasons outlined above.

# Reviewer report: Reviewer response for version 3

**DOI:** 10.5256/f1000research.169472.r306970

**Published:** 2024-08-16

**Authors:** Mingyan Cai

**Affiliations:** 1 Endoscopy Center, Zhongshan Hospital, Fudan University, Shanghai, Shanghai, China

No further comments

Are enough details provided of any physical examination and diagnostic tests, treatment given and outcomes?

Partly

Is the case presented with sufficient detail to be useful for other practitioners?

Partly

Is sufficient discussion included of the importance of the findings and their relevance to future understanding of disease processes, diagnosis or treatment?

Yes

Is the background of the case’s history and progression described in sufficient detail?

Yes

Reviewer Expertise:

Endoscopy

I confirm that I have read this submission and believe that I have an appropriate level of expertise to confirm that it is of an acceptable scientific standard.

# Reviewer report: Reviewer response for version 2

**DOI:** 10.5256/f1000research.159125.r302670

**Published:** 2024-07-18

**Authors:** Ali Bilal Ulas

**Affiliations:** 1 Ataturk University, Erzurum, Turkey

The article presents a case report of a child with a magnetic foreign body lodged in the esophagus, highlighting the difficulties faced in rural areas, such as limited equipment and manpower, and the need for innovative solutions.

 While the statement ‘the lower esophagus is the most common site of foreign body lodging’ is incorrect, it is important to evaluate the method proposed in the article beyond its content. The technique, which is considered an innovation, makes a statement that is contrary to established medical practice.

 The modified endoscopic hook technique is not an innovation, and it is neither suitable nor safe. Primum non nocere (First, do no harm)! Patient safety must remain the highest priority. I appreciate the challenges the authors faced and their good intentions in attempting to avoid perforation with a magnet. However, using a hook in the esophagus will undoubtedly cause more serious complications, such as damage to the trachea, aorta, and other organs, or mediastinitis, which will inevitably lead to significant outcomes. It is imperative that any unnecessary interventions that could lead to malpractice and compromise patient safety are avoided.

 The authors suggest repeating the procedure if it does not work on the first attempt. As thoracic surgeons, we strongly advise against repeated attempts due to the risk of iatrogenic esophageal perforations, which can cause serious complications. The article does not sufficiently discuss potential risks or complications associated with the modified tool. It is imperative to question why a hook for extracting foreign bodies in the esophagus is not available. Serious mortality and significant mortal consequences are associated with such interventions.

 Furthermore, the authors confirm that if the case had been in an urban area, a flexible endoscope would have been used. However, the authors were mistakenly concerned about urgent intervention for the esophageal SINGLE magnet. Even in centers with rigid and flexible endoscopes, multiprong forceps, hinged types of forceps, basket retrieval forceps, net retrieval devices, snares, high patient volume, and experienced hands, there is a possibility of complications. It would be more appropriate to perform such interventions in centers with multidisciplinary teams that can manage any complications that may arise.

 In conclusion, although the authors' intentions are understood and appreciated, the proposed method is not safe or advisable.

Are enough details provided of any physical examination and diagnostic tests, treatment given and outcomes?

Partly

Is the case presented with sufficient detail to be useful for other practitioners?

No

Is sufficient discussion included of the importance of the findings and their relevance to future understanding of disease processes, diagnosis or treatment?

No

Is the background of the case’s history and progression described in sufficient detail?

Partly

Reviewer Expertise:

Thoracic Surgery

I confirm that I have read this submission and believe that I have an appropriate level of expertise to state that I do not consider it to be of an acceptable scientific standard, for reasons outlined above.

# Response

**Published:** 2024-07-21

**Authors:** hamsu kadriyan

**Affiliations:** Faculty of Medicine, University of Mataram, Indonesia

Dear Reviewer,

 Thank you on reviewing my article.

1. The statement regarding the commonest site of FB lodging was change to “as the common site of FB lodging. The most common lodging site is upper part of esophagus” to prevent the misunderstandings.
2. To better understand the safety nature of this innovation, the authors have added the schematic picture of the innovation (Figure 2B). Therefore, it can be used more safely.
3. The potential impact of using hooks has been mention in paragraph 4 on the discussion heading.
4. If FB extraction fails with this method, the authors rely on the patient to be referred to a better hospital. (Paragraph 3 and 4 on the discussion heading)
5. The authors also believe that with this case report, we cannot definitively determine the safety and efficacy of this innovation. Therefore, the authors propose that better studies with a better methods may be conducted in the future to prove its safety and efficacy properties (last paragraph of the discussion title).

# Reviewer report: Reviewer response for version 2

**DOI:** 10.5256/f1000research.159125.r225008

**Published:** 2023-12-15

**Authors:** Dian Adi Syahputra

**Affiliations:** 1 Department of Surgery, Syiah Kuala University, Banda Aceh, Indonesia

Thank you for the revision.

 Suggestions to the author are mainly regarding the discussion of oesophageal anatomy and the most common site of foreign body impaction.

 Reference 10 states that the most common site is the upper esophagus at around 50%, contrary to the author's statement in the discussion section that it is the lower esophagus.

 The reference below supports the statement that the upper esophagus is the most common site. Please be very careful in writing statements in declared articles. Thank you.

 Shruti Jayachandra, Guy D. Eslick,

 A systematic review of paediatric foreign body ingestion: Presentation, complications, and management,

 International Journal of Pediatric Otorhinolaryngology,

 Volume 77, Issue 3,

 2013,

 Pages 311-317,

 ISSN 0165-5876,

 https://doi.org/10.1016/j.ijporl.2012.11.025.

 (https://www.sciencedirect.com/science/article/pii/S0165587612006519)

Are enough details provided of any physical examination and diagnostic tests, treatment given and outcomes?

Yes

Is the case presented with sufficient detail to be useful for other practitioners?

Yes

Is sufficient discussion included of the importance of the findings and their relevance to future understanding of disease processes, diagnosis or treatment?

Partly

Is the background of the case’s history and progression described in sufficient detail?

Yes

Reviewer Expertise:

Pediatric Surgeon

I confirm that I have read this submission and believe that I have an appropriate level of expertise to confirm that it is of an acceptable scientific standard.

# Reviewer report: Reviewer response for version 1

**DOI:** 10.5256/f1000research.142254.r161310

**Published:** 2023-11-15

**Authors:** Dian Adi Syahputra

**Affiliations:** 1 Department of Surgery, Syiah Kuala University, Banda Aceh, Indonesia

A beneficial case report on managing FB in areas with limited resources.

 There are some comments to improve this case report to make it better for readers.

1. The author should not use the term "otolaryngologists" in this case report, especially in the abstract and the last paragraph of the discussion. It is better to use global terms such as doctors or physicians.
2. In the discussion section, the author should discuss the anatomy of the esophagus, which results in the retention of the FB in the esophagus to the stomach.
3. The author should refrain from doing self-citation in this report because there are 2 self-citations in the references. Many case reports discuss FB in children, especially in the esophagus.

Are enough details provided of any physical examination and diagnostic tests, treatment given and outcomes?

Yes

Is the case presented with sufficient detail to be useful for other practitioners?

Yes

Is sufficient discussion included of the importance of the findings and their relevance to future understanding of disease processes, diagnosis or treatment?

Partly

Is the background of the case’s history and progression described in sufficient detail?

Yes

Reviewer Expertise:

Pediatric Surgeon

I confirm that I have read this submission and believe that I have an appropriate level of expertise to confirm that it is of an acceptable scientific standard, however I have significant reservations, as outlined above.

# Response

**Published:** 2023-11-19

**Authors:** hamsu kadriyan

**Affiliations:** Faculty of Medicine, University of Mataram, Indonesia

Dear reviewer,

 Thank you for your comment on my article. Regarding your suggestion, I have revised the article as follows:

 1. I have changed the word "otolaryngologist" to "physician".

 2. I have added the anatomy of the esophagus in the discussion section.

 3. To prevent self-citation I deleted one of the references, but the other one is significant to support the explanation of health services problem in a rural area. I also added some other references to support the importance of this manuscript.

# Reviewer report: Reviewer response for version 1

**DOI:** 10.5256/f1000research.142254.r165757

**Published:** 2023-11-15

**Authors:** Mingyan Cai

**Affiliations:** 1 Endoscopy Center, Zhongshan Hospital, Fudan University, Shanghai, Shanghai, China

The authors described a modified endoscopic hook for extracting magnetic esophageal foreign body. It has some novelty, but such big magnetic foreign body will not always successfully hooked by the small hook. The reproduction of the result is limited in other cases, since the hook itself sometimes won't have enough power to extract the foreign body from the narrowing space of the esophageal entrance.

 GI endoscopy with accessary channels through which can apply a series of accessories has more advantages than this rigid scope.

Are enough details provided of any physical examination and diagnostic tests, treatment given and outcomes?

Partly

Is the case presented with sufficient detail to be useful for other practitioners?

Partly

Is sufficient discussion included of the importance of the findings and their relevance to future understanding of disease processes, diagnosis or treatment?

Yes

Is the background of the case’s history and progression described in sufficient detail?

Yes

Reviewer Expertise:

Endoscopy

I confirm that I have read this submission and believe that I have an appropriate level of expertise to state that I do not consider it to be of an acceptable scientific standard, for reasons outlined above.

# Response

**Published:** 2023-11-19

**Authors:** hamsu kadriyan

**Affiliations:** Faculty of Medicine, University of Mataram, Indonesia

Dear Reviewer

 Thank you for your comment on my manuscript.

 The setting of this case was in a rural area. If the case was found in an urban area, I agree to the use of a flexible endoscope. To support the importance of this innovation in rural areas, I have inserted some references in the discussion section
